# 5-Cog Study: Cross-Cultural Comparison of Subjective Cognitive Complaints in a Diverse Primary Care Population

**DOI:** 10.1093/geroni/igaa057.1162

**Published:** 2020-12-16

**Authors:** Emmeline Ayers, Erica F Weiss, Joe Verghese

**Affiliations:** 1 Albert Einstein College of Medicine, Bronx, New York, United States; 2 Montefiore Medical Center, Bronx, New York, United States

## Abstract

Subjective cognitive complaints (SCC) are risk factors for cognitive decline in older adults. A link between SCC and depressive symptoms has also been reported. These associations have not been much studied in non-White populations. We examined the relationship of SCC with cognitive function and depressive symptoms in adults aged 65 and older attending a primary care clinic in the Bronx. Five common SCC questions (four memory-related and one non-memory-related) were identified by literature review. Linear regressions, adjusted for age, sex and education years, were used to examine associations between individual SCC and cognitive function (Montreal Cognitive Assessment (MoCA) score and Hopkins Verbal Learning Test (HVLT) recall score) and depressive symptoms (Geriatric Depression Scale (GDS) score) for Hispanic (n=53) and non-Hispanic Black (n=47) adults. Mean number of SCC was similar for Blacks and Hispanics (2.3 vs. 2.4, p=0.752). Hispanics performed worse on the MoCA than Blacks (16.4 vs. 18.5, p=0.012), but education explained this difference. GDS and HVLT were similar across groups. For Hispanics only, a response of fair or poor to the question “how is your memory for a person your age?” was associated with worse MoCA scores (β -2.6; p=0.008). SCC were not associated with HVLT scores for either group. Four SCC for Blacks and two for Hispanics were associated with worse GDS scores. In an urban clinic population, SCC for Blacks and Hispanics were associated more with depressive symptoms than cognition. Further research is needed to identify SCC that better correlate with cognitive function in diverse populations.

